# Effects of DPP-4 Inhibitors on the Heart in a Rat Model of Uremic Cardiomyopathy

**DOI:** 10.1371/journal.pone.0027861

**Published:** 2011-11-18

**Authors:** Lyubov Chaykovska, Karoline von Websky, Jan Rahnenführer, Markus Alter, Susi Heiden, Holger Fuchs, Frank Runge, Thomas Klein, Berthold Hocher

**Affiliations:** 1 Charité - Universitätsmedizin Berlin, Center for Cardiovascular Research, Institute for Pharmacology and Toxicology, Berlin, Germany; 2 Institute of Nutritional Science, University of Potsdam, Potsdam-Nuthetal, Germany; 3 Charité - Universitätsmedizin Berlin, Medizinische Klinik für Endokrinologie und Nephrologie, Berlin, Germany; 4 Boehringer Ingelheim Pharma GmbH & Co. KG, Biberach an der Riss, Germany; Virginia Commonwealth University, United States of America

## Abstract

**Background:**

Uremic cardiomyopathy contributes substantially to mortality in chronic kidney disease (CKD) patients. Glucagon-like peptide-1 (GLP-1) may improve cardiac function, but is mainly degraded by dipeptidyl peptidase-4 (DPP-4).

**Methodology/Principal Findings:**

In a rat model of chronic renal failure, 5/6-nephrectomized [5/6N] rats were treated orally with DPP-4 inhibitors (linagliptin, sitagliptin, alogliptin) or placebo once daily for 4 days from 8 weeks after surgery, to identify the most appropriate treatment for cardiac dysfunction associated with CKD. Linagliptin showed no significant change in blood level AUC(0-∞) in 5/6N rats, but sitagliptin and alogliptin had significantly higher AUC(0-∞) values; 41% and 28% (*p = *0.0001 and *p = *0.0324), respectively. No correlation of markers of renal tubular and glomerular function with AUC was observed for linagliptin, which required no dose adjustment in uremic rats. Linagliptin 7 µmol/kg caused a 2-fold increase in GLP-1 (AUC 201.0 ng/l*h) in 5/6N rats compared with sham-treated rats (AUC 108.6 ng/l*h) (*p = *0.01). The mRNA levels of heart tissue fibrosis markers were all significantly increased in 5/6N vs control rats and reduced/normalized by linagliptin.

**Conclusions/Significance:**

DPP-4 inhibition increases plasma GLP-1 levels, particularly in uremia, and reduces expression of cardiac mRNA levels of matrix proteins and B-type natriuretic peptides (BNP). Linagliptin may offer a unique approach for treating uremic cardiomyopathy in CKD patients, with no need for dose-adjustment.

## Introduction

Chronic kidney disease (CKD) and moreover, end-stage renal disease (ESRD), have been shown to increase cardiovascular disease and risk of death [1], [2]. This has been substantiated in a systematic review on mortality risk, which concluded that increased risk for all-cause mortality in CKD patients was largely driven by cardiovascular deaths (58% deaths from 13 studies reporting both cardiovascular and all-cause deaths) [3].

Glucagon-like peptide-1 (GLP-1) is an incretin hormone secreted by the small intestine in response to nutrient ingestion. Although the major physiological function of GLP-1 appears to relate to glycaemic control, evidence suggests that GLP-1 plays an important role in the cardiovascular system. GLP-1 receptors (GLP-1Rs) are expressed in the heart and vasculature of rodents as well as humans. Research has shown that GLP-1R agonists affect a wide range of cardiovascular parameters, including heart rate, blood pressure, vascular tone and myocardial contractility. Importantly, these agents may also have beneficial effects in the setting of cardiovascular disease (CVD). For example, GLP-1 has been found to exert cardioprotective actions in experimental models of dilated cardiomyopathy, hypertensive heart disease and myocardial infarction (MI). Preliminary clinical studies also suggest that GLP-1 infusion may improve cardiac contractile function in chronic heart failure patients with and without diabetes, and in MI patients after successful angioplasty [4], [5], [6]. However, the cardiovascular effects of a pharmacological increase in GLP-1 in patients with CKD have not been determined.

Dipeptidyl peptidase-4 (DPP-4) inhibitors are considered incretin enhancers, because they inhibit the enzymatic degradation of incretins, in particular, GLP-1 [7] and therefore are established therapies for type 2 diabetes. At the same time, DPP-4 inhibition does not cause hypoglycemia, as was previously shown by Bergman et al in a study in healthy male volunteers [8]. Because the action of GLP-1 on insulin secretion is strictly glucose dependent, the risk of hypoglycaemia associated with DPP-4 inhibitors is low [9].The main elimination route of the first generation of approved DPP-4 inhibitors (sitagliptin, saxagliptin, vildagliptin) is via the kidney [7], [10]. Dose adjustment in patients with diabetes and chronic renal failure (CRF) is thus necessary [10], [11]. Linagliptin a recently launched DPP-4 inhibitor is different in this respect with primary elimination via the bile (approximately 85% of the orally administered dose) and only 1–5% eliminated via the urine [12], [13].

We studied the pharmacokinetics and pharmacodynamics of different DPP-4 inhibitors, in the settings of CRF, in order to determine the properties of DPP-4 inhibitors to be used in patients with impaired renal function, and investigated the effects of linagliptin on biomarkers of cardiac and renal fibrosis. The results showed that DPP-4 inhibition increases plasma GLP-1 levels, particularly in uremia, suggesting that linagliptin may offer a unique approach for treating uremic cardiomyopathy in CKD patients.

## Results

This study showed that 5/6N caused a significant decrease in GFR as measured by creatinine clearance and increased plasma cystatin C levels. Tubular function was significantly impaired after 5/6N as evidenced by increased plasma β2-microglobulin, NGAL and osteopontin levels. There was no significant difference in DPP-4 activity in 5/6N rats compared with sham-operated rats before treatment (Table 1), but DPP-4 activity decreased significantly in all groups following drug administration with no significant differences between control or 5/6N groups. The strongest DPP-4 inhibition was achieved after administration of 7 µmol/kg linagliptin (Table 2), whereas the other groups were comparable. The GLP-1 receptor mRNA expression was reduced about 40% in uremic rats as compared to healthy control rats (table 1).

**Table 1:** Characteristics of the model of CRF: kidney function parameters, cardiac histology.

Kidney function parameters	Sham-operated rats	5/6 N rats
Endogenous creatinine clearance (ml/24 h/g BW, mean ± SEM)	4.45±1.5	3.27±0.1***
Cystatin C (ng/ml, mean ± SEM)	700±35.7	1434±77.6***
β_2_-microglobulin (µg/ml, mean ± SEM)	20.4±2.4	33.3±1.34***
NGAL (ng/ml, mean ± SEM)	286±23	680±56.3***
Osteopontin (ng/ml, mean ± SEM)	33.5±3.2	56.1±6.2***
Relative left ventricular weight (left ventricular weight/body weight) (%± SEM)Cardiac interstitial fibrosis (% of section ± SEM)Myocyte diameter (*µ*m ± SEM)GLP-1 receptor mRNA in heart tissue	0.20±0.012.1±0.111.8±1.11.12±0.19	0.29±0.03**2.5±0.1*17.1±2.0**0.75±0.15 ***
DPP-4 activity(fluorescence units, mean ± SEM)	84315.77±3844.56	80594.24±3640.53

Values are given in mean±SEM; sham-operated rats group, 5/6N rats, two-tailed students t-test.

*p*<*0.05;

**p*<*0.01;

***p*<*0.001.

**Table 2:** DPP-4 activity.

	Control	Linagliptin 0.5 µmol/kg mean±SEM	Linagliptin 7 µmol/kg mean±SEM	Sitagliptin 7 µmol/kg mean±SEM	Alogliptin 7 µmol/kg mean±SEM
DPP-4 activity	82156±20673	45951±13622	21019±5152	57219±21531	50874±8958

DPP-4 activity was measured 24 h after administration of the respective compounds (N* = *13–17 per DPP-4 inhibitor group, control N* = *57; no difference was made between sham-operated rats and 5/6N groups). All drugs showed a significant inhibition of DPP-4 activity (*p<*0.001) compared with control.

We tested plasma glucose concentrations on alternate days while the rats were being treated with DPP-4 inhibitors. There was no change in plasma glucose in rats treated with DPP-4 inhibitors compared with nontreated animals (data not shown).

### Influence of DPP-4 inhibitor administration on pharmacokinetic parameters in sham-operated rats and 5/6N rats

There were no difference in the blood concentration of linagliptin 0.5 µmol/kg in 5/6N rats (AUC(0-∞) 257.5±21.44 nmol•h/l; *p = *0.771) compared with sham-operated rats (AUC(0-∞) 267.4±28.85 nmol•h/l). A similar effect was seen after administration of linagliptin 7 µmol/kg in 5/6N rats (AUC(0-∞) 1252±372.8 nmol•h/l) compared with sham-operated rats (AUC(0-∞) 748±74.52 nmol•h/l), with a slight, but not significant (*p = *0.283), decrease in AUC levels observed for linagliptin. In contrast, both sitagliptin and alogliptin (7 µmol/kg) had a significantly higher AUC(0-∞) in 5/6N rats compared with sham-operated rats: 41% and 28% (*p = *0.0001 and *p = *0.0324), respectively: sitagliptin sham-operated rats, AUC(0-∞) 3690±103 nmol•h/l; 5/6N rats, AUC(0-∞) 6238±423 nmol•h/l and alogliptin sham-operated rats, AUC(0-∞) 1771±225.5 nmol•h/l; 5/6N rats, AUC(0-∞) 2445±166.6 nmol•h/l) (Table 3).

**Table 3:** AUC of different DPP-4 inhibitors in sham-operated rats vs 5/6N rats.

Treatment	Sham-operated rats, (AUC_(0-∞)_), nmol•h/lmean±SEM	5/6N rats (AUC_(0-∞)_), nmol•h/lmean±SEM
Alogliptin 7 µmol/kg	1771±225.5	2445±166.6 *
Linagliptin 0.5 µmol/kg	267.4±28.85	257.5±21.44
Linagliptin 7 µmol/kg	1252±378.8	748.2±74.52
Sitagliptin 7 µmol/kg	3690±103	6238±423 ***

*N = *5–6 sham-operated rats and 8–13 5/6N rats.

*p<0.05,

***p<0.001.

No correlation of markers of renal tubular and glomerular function with AUC for linagliptin was observed. In contrast, sitagliptin AUC significantly correlated with GFR, cystatin C, β2-microglobulin and NGAL, but not with osteopontin. Alogliptin AUC correlated significantly with cystatin C, β2-microglobulin and osteopontin (*p<*0.05), but not with GFR and NGAL (Table 4).

**Table 4:** Correlation between AUC of different DPP-4 inhibitors and kidney function parameters in 5/6N rats.

Kidney function parameters	Alogliptin7 µmol/kg, r^2^	Sitagliptin7 µmol/kg, r^2^	Linagliptin0.5 µmol/kg, r^2^	Linagliptin7 µmol/kg, r^2^
GFR	0.211	0.374*	0.098	0.18
Cystatin C	0.376*	0.499**	0.004	0.189
β_2_-microglobulin	0.391*	0.543**	0.001	0.092
NGAL	0.295	0.604**	0.091	0.1
Osteopontin	0.406*	0.325	0.005	0.154

Correlation analysis between DPP-4 inhibitors and kidney function parameters was performed using Spearman's rank correlation test. *N = *6–12 per group.

*p<0.05,

**p<0.01.

### Influence on pharmacodynamic parameters in 5/6N rats following DPP-4 inhibitor administration

To gain further insight into whether kidney function is changed in the setting of 5/6N after short-term administration of DPP-4 inhibitors, we investigated the tubular and glomerular markers following drug administration for 4 days and compared these with the respective pre-treatment values. Creatinine clearance was not changed after DPP-4 inhibition, independently of the substance used for treatment (data not shown). The glomerular marker, cystatin C, was significantly increased following 4 days of treatment with alogliptin and unchanged after sitagliptin treatment. Linagliptin showed a trend toward decreasing cystatin C level. None of the drugs affected NGAL levels. Sitagliptin further significantly aggravated tubular injury by increasing the concentration of β2-microglobulin. This marker remained unchanged by the other compounds. At the same time, sitagliptin (7.0 µmol/kg [*p<*0.01]), alogliptin (7.0 µmol/kg [*p<*0.01]) and linagliptin (0.5 µmol/kg [*p<*0.001]) significantly reduced the concentration of osteopontin, a marker of tubular injury and fibrosis. The effect of the higher dose of linagliptin pointed in the same direction, but did not reach statistical significance (for details see Table 5).

**Table 5:** Effects of the different DPP-4 inhibitors on markers of kidney function before and after drug administration in 5/6N rats.

Treatment	Condition	Cystatin C [ng/ml] mean±SEM	β_2_-microglobulin [µg/ml] mean±SEM	NGAL [ng/ml] mean±SEM	Osteopontin[ng/ml] mean±SEM
Alogliptin 7.0 µmol/kg	before	1015.7±80.1	32.5±1.5	473.8±68.5	50.9±5.5
	after	1190.3±103.7*	32.3±2.2	520.4±58.3	39.1±8.8**
Linagliptin0.5 µmol/kg	before	1731.1±53.4	35.2±1.6	921.0±117.6	70.9±4.7
	after	1564.4±60.6	30.3±1.5	821.2±53.1	48.1±3.8***
Linagliptin7.0 µmol/kg	before	1633.6±177.9	34.2±3.9	753.5±91.7	54.2±10.0
	after	1613.3±180.7	36.8±5.2	804.0±128.8	49.4±10.7
Sitagliptin7.0 µmol/kg	before	1192.0±71.5	30.4±0.9	490.3±93.7	48.5±4.4
	after	1233.6±105.6	39.7±3.8*	1394.5±397.4	33.4±2.4**

Indicated kidney markers were analyzed in blood before and after (4 days + 72 h) DPP-4 inhibitor administration in 5/6N rats. (*N = * 4–6 in sham-operated rats and 6–12 in 5/6N rats).

*p<0.05;

**p<0.01;

***p<0.001.

### Influence of linagliptin treatment on gene expression of left ventricular dysfunction marker, BNP, and of fibrotic markers in 5/6N rat heart

**Figure 1:**
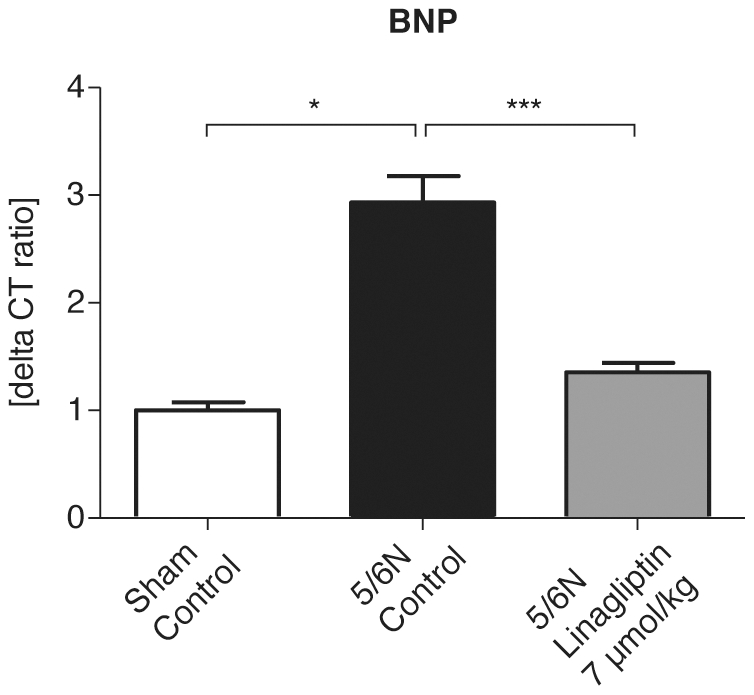
Experimental design.

**Figure 2:**
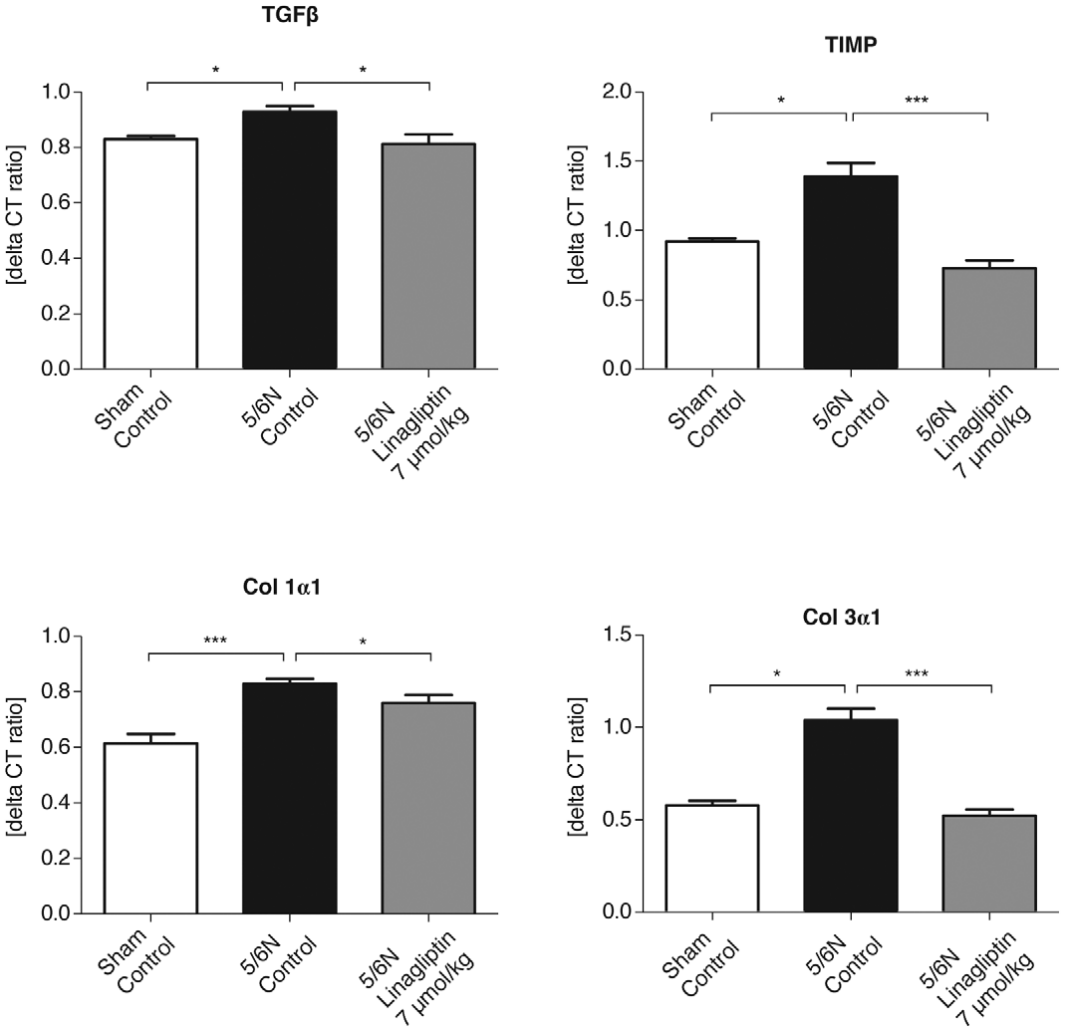
mRNA expression of BNP in uremic rat heart. Gene expression of the marker of left ventricular dysfunction BNP was significantly increased in rats after initiation of uremia. Treatment with linagliptin at a dose of 7 µmol/kg significantly reduced mRNA expression of BNP in uremic rat heart. Values are given in mean ± SEM. *N = *7 sham-operated rats, 5 5/6N rats and 12 5/6N linagliptin-treated rats. *p<0.05; ***p<0.001.

Based on the pharmacodynamic data described above, we have selected linagliptin as the most suitable and safest drug for further efficacy studies in rats. We found a significant increase in mRNA expression of BNP, TGF-β1, TIMP-1, Col1α1 and Col3α1 in uremic rat heart compared with sham-operated rat heart (see Figures. 1, 2). Moreover, treatment of the 5/6N rats for only 4 days with linagliptin (7 µmol/kg) significantly reduced gene expression of BNP and all investigated fibrosis markers (Figure 1; 5/6N linagliptin 7 µmol/kg) almost to baseline levels of healthy control rats. C_max_ values were significantly (*p = *0.03) higher for 5/6N (6.4±2.6 pg/ml) vs sham animals (3.9±1.9 pg/ml). No significant changes in DPP-4 inhibition were detected between sham and 5/6N animals (data not shown).

## Discussion

The overall goal of the present study was to compare the pharmacokinetic properties of available DPP-4 inhibitors in a rat model of uremic heart disease and select the optimal compound based on these data for the first pharmacodynamics analyses of potential efficacy in this rat model.

We have shown that renal impairment does not affect the pharmacokinetics of linagliptin, whereas it increases the exposure of sitagliptin and alogliptin. In the present study, only linagliptin was found not to further aggravate pathological changes of glomerular and tubular markers in rats with CRF, suggesting that it is a safe approach to be used in patients with CRF. Consequently, linagliptin was also the compound of choice to investigate further effects on uremic cardiomyopathy. This is of potential clinical impact, since patients with advanced stages of renal impairment are characterized by a high overall cardiac morbidity and mortality.

Our study demonstrated for the first time that short-term treatment with all DPP-4 inhibitors (linagliptin, sitagliptin and alogliptin) decreases the plasma concentration of the vascular calcification marker, osteopontin (Table 5). This suggests a class effect also, because among all biomarkers investigated only osteopontin was consistently reduced by DPP-4 inhibitors. The effect did not reach significance in the higher dose of linagliptin, most likely due to the high variability of osteopontin data in this group, however, also those data point towards reduced osteropontin levels.

Osteopontin is known to be associated with vascular calcification and cardiovascular morbidity in humans [14]. It would be of major clinical interest to see whether the osteopontin lowering effect of DPP4 inhibitors can be seen likewise in the ongoing clinical trials using compounds of this new class.

In addition, linagliptin administration decreased cardiac mRNA levels of BNP—a marker of left ventricular dysfunction (Figure 1), and reduced cardiac mRNA expression of fibrosis markers, such as TGF-β1, TIMP-1, Col1α1 and Col3α1 in uremic rats (Figure 2) to baseline levels.

The 5/6N rat model of CRF with elimination of two-thirds of the left kidney after previous right nephrectomy is a gold standard for the study of CKD. Its pathological characteristics resemble those of renal failure in humans [15] and are widely used for investigation of pharmacokinetics of different compounds in the setting of renal impairment [16], [17]. 

We have shown a simultaneous increase in plasma concentration of both renally-eliminated DPP-4 inhibitors (sitagliptin and alogliptin) and markers of glomerular and tubular injury (Table 4). Only the AUC of linagliptin remained unchanged in the setting of CRF, which strongly suggests that linagliptin is the only DPP-4 inhibitor that does not require dose adjustment in patients with CRF.

Investigating the influence of DPP-4 inhibition on kidney function, we revealed that treatment of rats with DPP-4 inhibitors does not influence GFR, a finding that agrees with the work of Kirino *et al*. [18], who showed no significant differences in serum creatinine and creatinine clearance levels between wild-type and DPP-4-deficient mice. Cystatin C was previously shown as a more sensitive and more efficient diagnostic marker of kidney function compared with serum creatinine [19], [20], [21]. Plasma cystatin C level was increased in rats treated with alogliptin (Table 5), suggesting that alogliptin administration caused a deterioration in kidney function, and administration of sitagliptin caused a significant increase in the concentration of the tubular injury marker, β_2_-microglobulin, in 5/6N rats. Only linagliptin administration did not further aggravate a decline in kidney function in 5/6N rats, suggesting that it is a safe medication to be administered in the settings of CKD.

It is well known that 5/6N leads to uremic cardiomyopathy [22], [23], [24], where transforming growth factor β (TGF-β), tissue inhibitor of matrix metalloproteinases (TIMP-1) and collagen (Col3α1) are increased in the uremic heart [25], [26], [27]. Inhibitors of these factors have antifibrotic properties, and ameliorate pathologic changes in the heart in the CRF setting [26], [27]. DPP-4 was previously reported as one of the factors that promotes tissue fibrosis [28]; we have shown that all investigated DPP-4 inhibitors (linagliptin, sitagliptin and alogliptin) decrease plasma concentrations of the fibrosis marker, osteopontin (Table 5), which has recently been called “the killer of patients with CKD” [29], due to its role in vascular calcification. A link between diabetes, DPP-4 inhibitors and osteopontin was described by Senkel et al. in their study on hepatocyte nuclear factor 1β (HNF1β) [30]; the HNF1β promoted gene expression of both targets DPP-4 and osteopontin. In light of these data, it is of interest that short-term treatment of uremic rats with the DPP-4 inhibitor, linagliptin, normalizes the mRNA expression of all of the key factors of uremic cardiomyopathy (transforming growth factor β (TGF-β), tissue inhibitor of matrix metalloproteinases (TIMP-1) and collagen (Col3α1) to baseline level (Figure 2).

Previous studies have already reported a link between DPP-4 inhibition and improvement in cardiac function. DPP-4-deficient rats had a better preservation of cardiovascular function than wild-type rats during endotoxemia, which correlated with a more prominent elevation of GLP-1 signaling. These findings coincided with the pretreatment of the GLP-1 analogue, exendin-4, where the deterioration of cardiovascular function during endotoxemia was significantly reversed in wild-type rats [31]. Elevation of GLP-1 by DPP-4 inhibitors may have emerging cardiovascular effects in uremic heart disease.

**Figure 3:**
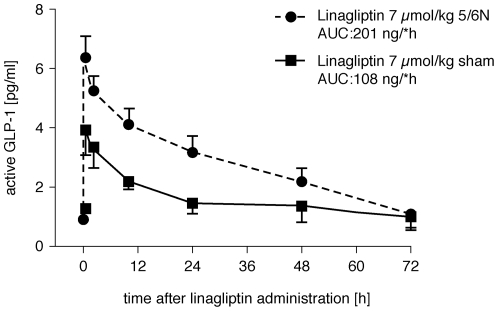
Gene expression of fibrosis markers in uremic rat heart. Uremic condition led to a significant increase in gene expression of profibrotic factors TGF-β1, TIMP-1, Col1α1 and Col3α1 in the 5/6 nephrectomized rats compared with corresponding sham-operated rats. Linagliptin at a dose of 7 µmol/kg significantly inhibited mRNA expression of profibrotic factors TGF-β1, TIMP-1, Col1α1 and Col3α1 in uremic rat heart. Values are given in mean ± SEM. *N = *7 sham-operated rats, 5 5/6N rats, and 12 5/6N linagliptin-treated rats. *p<0.05; ***p<0.001.

Baseline GLP-1 concentrations in non-fasted rats with and without renal failure are low and almost similar (Figure 3). However, treatment with linagliptin 7 µmol/kg caused a 2-fold increase in GLP-1 (AUC 201.0 ng/l*h) in 5/6N rats compared with sham-treated rats (AUC 108.6 ng/l*h) (*p = *0.01). These findings are in line with a recent clinical study [32]. The better efficacy of the DPP-4 inhibitor, linagliptin, in uremic rats with respect to plasma GLP-1 concentrations has two implications:

- DPP-4 inhibition as novel treatment of T2D in patients with impaired renal function might be particularly effective. In patients with normal kidney function, treatment with DPP-4 inhibitors usually results in somewhat less pronounced elevation of GLP-1 as compared with the direct administration of synthetic GLP-1 [33]; however, this hypothesis needs to be tested in further clinical trials.
- The pronounced elevation of GLP-1 after treatment with linagliptin in uremic rats might contribute to the remarkable effects of linagliptin on cardiac matrix synthesis via direct cardiac GLP-1 receptor mediated effects. However, it needs to be considered that cardiac GLP-1 receptor mRNA expression was lower in uremic rats (table 1) as compared to wild type rats. Our data with respect to the plasma biomarker osteopontin (table 5) as well as the cardiac mRNA expression of BNP and fibrosis biomarkers (figures 1 and 2) seem to indicate that the net effect of GLP-1 elevation after DPP4 inhibition with Linagliptin results in cardio-protection. However, this needs to be proven in long-term studies (see also study limitation section below).

The underlying mechanism for this pronounced effect of DPP-4 inhibition on blood GLP-1 concentrations under the condition of impaired kidney function is most likely related to the renal clearance of GLP-1, which is impaired in renal failure, and the concomitant inhibition of its degradation by DPP-4. This hypothesis needs to be confirmed by controlled studies that would investigate the influence of active and total GLP-1 in healthy and renal-insufficient animals treated with DPP-4 inhibitors.

Although the major physiological function of GLP-1 appears to be in relation to glycemic control, there is growing evidence to suggest that it plays an important role in the cardiovascular system [6]. GLP-1 receptors (GLP-1Rs) are expressed in the heart and vasculature, and recent studies have shown that GLP-1 receptor agonists have cardiovascular actions, independent of improving glucose homeostasis, such as modulation of heart rate, blood pressure, vascular tone and myocardial contractility. Importantly, it appears that these agents may also have beneficial effects in the setting of cardiovascular disease, eg, GLP-1 has been found to exert cardioprotective effects in experimental models of dilated cardiomyopathy, hypertensive heart failure and myocardial infarction (MI). Preliminary data of clinical studies also indicated that GLP-1 infusion may improve cardiac contractile function in chronic heart failure (CHF) patients with and without diabetes, and in MI patients after successful angioplasty [6].

It is of particular note that the transcription levels of BNP decreased to baseline levels after treatment with the DPP-4 inhibitor, linagliptin (Figure 1). BNP is a biomarker of acute and CHF also in renally compromised patients. Its levels are elevated in patients with left ventricular dysfunction. Rapid changes in BNP levels (up to 30% during the first 24 hours of treatment) reflect an adequate response to CHF therapy [34].

**Figure 4:**
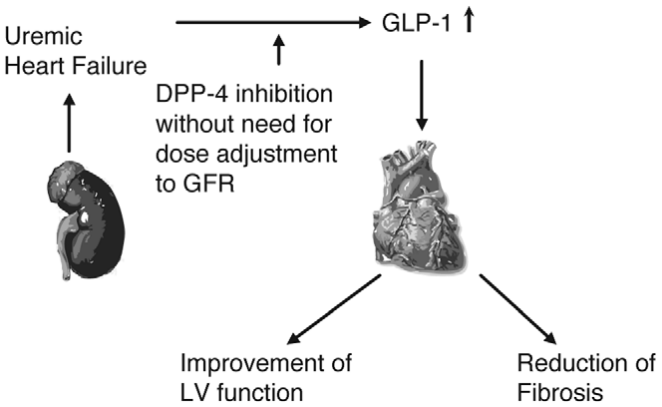
Influence of DPP-4 inhibition on cardiac impairment in the setting of uremia.

In our study, brain-derived natriuretic peptide mRNA was detected and was increased in the cardiac tissue of 5/6N rats and decreased after short-term treatment of uremic rats with linagliptin, suggesting an immediate improvement in cardiac function after DPP-4 inhibition (Figure 1, Figure 4). In addition, we have shown an inhibition of gene expression of profibrotic factors TGF-β1, TIMP-1, Col3α1 and Col3α1 in the uremic rat heart after DPP-4 inhibitor treatment.

This is the first study showing that the DPP-4 inhibitor, linagliptin, may exert positive effects on CHF in the setting of uremia (Figure 4).

It is a clear study limitation that the treatment was pretty short. This forced us to evaluate potential cardiac efficiasy in the 5/6 nephrectomy model based on biomarkers like osteopontin, elevation of plasma GLP-1, cardiac expression of BNP mRNA and cardiac mRNA of TGF-β1, TIMP-1, Col1α1 as well as Col3α1. A further limitation of this study is the fact that functional readouts of heart function like echocardiography were not performed in the current study. Our study should stimulate studies aiming to analyses more longterm treatment effects and the potential translation of this treatment into improvement of mortality in this model of uremic cardiomyopathy. The potential antifibrotic effects of DPP-4 inhibitors could provide an additional benefit for patients with CKD and heart diseases that very often accompany T2D [35] and may provide new therapy options for this class of drugs.

Additional research could also be undertaken to evaluate the effects of the DPP-4 inhibitor linagliptin in 5/6 nephrectomized rats with direct GLP-1 infusions, and to compare doses of linagliptin with doses of GLP-1 infusions that lead to similar plasma concentrations. Such research could determine whether linagliptin, in addition to its GLP-1 elevating effect, blocks the degradation of other DPP-4 substrates with potential cardiac targets. In addition, research should be undertaken to investigate whether a combination of DDP-4 inhibitors and GLP-1 agonists potentiate cardiac efficacy. This has not yet been determined even in non-uremic cardiomyopathy models.

The non-renally eliminated DPP-4 inhibitor, linagliptin, has been shown in this rat model to be safe in the CRF setting. Linagliptin markedly increased plasma GLP-1 concentrations in uremic rats and decreased gene expression of BNP, a marker of left ventricular dysfunction, as well as the fibrotic markers TGF-β, TIMP-1, Col 1α1 and Col 3α1 in uremic rat heart.

Further investigation addressing long-term DPP-4 inhibition in the uremic rat heart is warranted to confirm possible new therapeutic applications for the treatment of CHF.

## Materials and Methods

This study was carried out in strict accordance with the recommendations in the Guide for the Care and Use of Laboratory Animals of the National Institutes of Health. The protocol was approved by the Committee on the Ethics of Animal Experiments of the city of Berlin, Germany (Landesamt für Gesundheit und Soziales (LAGeSo, Turmstr. 2l, D-10559 Berlin, Germany), permit number: G0366/08. All surgery was performed under inhalation anesthesia with isoflurane, and all efforts were made to minimize suffering.

### Experimental design

Rats in which two-thirds of the left kidney were surgically eliminated after previous right nephrectomy (5/6-nephrectomized [5/6N] rats) were randomly allocated into four groups (*N = *8–13 rats per group) according to the following oral pretreatment: linagliptin 0.5 µmol/kg/day corresponding to 0.24 mg/kg/day, linagliptin 7 µmol/kg/day (3.3 mg/kg/day), sitagliptin 7 µmol/kg/day (2.85 mg/kg/day), alogliptin 7 µmol/kg/day (2.34 mg/kg/day); all calculations based on the free bases. The 0.5-µmol/kg dose of linagliptin was selected as being the theoretical AUC equivalent to the 5-mg dose used in humans. Sham-operated rats were divided into four control groups (*N* = 5–6 rats per group) in the same manner.

**Figure 5:**
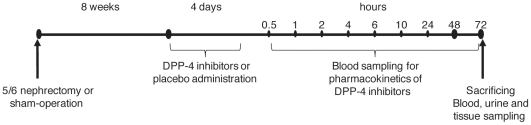
Active GLP-1 levels in uremic vs sham animals. The uremic situation resulted in a significant increase of GLP-1 AUC in 5/6N rats compared with controls. Linagliptin (7 µmol/kg) was administered daily for 4 days. Following the last dose, plasma was taken after the time points indicated and active GLP-1 was detected. Values are given as mean ± SEM. *N = *5 sham-operated, and 8-12 rats for 5/6N.

Commencing at 8 weeks after surgery, substances were administered once daily via oral gavage for 4 consecutive days. On the fourth day of treatment, blood samples were collected from the tail vein into ethylenediaminetetraacetic acid (EDTA)-coated vials at 0.5, 1, 2, 4, 6, 10, 24, 48 and 72 h after administration of the substance (Figure 5).

After centrifugation, plasma samples were stored at –20°C until bioanalytical measurement. After the last blood sampling, animals were sacrificed and kidneys were harvested and snap frozen in liquid nitrogen.

Studies of kidney function included measurements of serum and urine creatinine, glomerular filtration rate (GFR) and cystatin C at 8 weeks after surgery.

### Animal surgery

Animal procedures were approved by the local authorities and complied with the German Animal Protection Act. Male Wistar rats (250–290 g; Charles River Laboratories, Sulzfeld, Germany) were housed under standardized conditions with water and food ad libitum. All procedures were performed under inhalation anesthesia with isoflurane.

Chronic renal failure was initiated by a two-step 5/6 nephrectomy as previously described [36], [37]. Briefly, a right kidney was exposed via laparotomy and removed after ligation of the renal pedicle. After a 2-week recovery period, the left kidney was procured and lower and upper poles were surgically removed in such a way that one-third of the left kidney mass remained. Sham-operated rats underwent the same operations, but kidneys were only mobilized instead of being removed. After recovery from anesthesia, animals were transferred to the housing facility and monitored until sacrifice. Body weight and urine output were measured together with blood sampling at Week 8 after surgery. This study was carried out in strict accordance with the recommendations in the Guide for the Care and Use of Laboratory Animals of the National Institutes of Health. The protocol was approved by the Committee on the Ethics of Animal Experiments of the city of Berlin, Germany (Landesamt für Gesundheit und Soziales (LAGeSo, Turmstr. 2l, D-10559 Berlin, Germany), permit number: G0366/08. All surgery was performed under inhalation anesthesia with isoflurane, and all efforts were made to minimize suffering.

### Renal function tests

Induction of CRF by 5/6 nephrectomy was confirmed by measuring creatinine clearance and plasma cystatin C as markers of glomerular filtration. Creatinine concentration was measured by the Jaffe colorimetric method. Creatinine clearance was estimated as described previously [36]. Plasma concentrations of β2-microglobulin, neutrophil gelatinase-associated lipocalin (NGAL) and osteopontin were measured by automated immunoassays (Rules-Based Medicine, Inc., Austin, Texas) for the assessment of tubular function.

Cardiac histology. Tissue samples were all embedded in paraffin, cut into 3 µm and 1 µm sections and subjected to Sirius Red, HE and Elastica-van Gieson staining. Cardiac morphology (interstitial fibrosis, myocyte diameter) was analyzed using a computer-aided image analysis system (for details see: 26, 27, 28). Briefly, interstitial fibrosis was evaluated by estimating the relationship of the total section area to fibrotic section area. We analyzed 25 sections per animal. The myocyte diameter was investigated in 1 µm sections after HE staining exactly as described previously (26,27,28). We analyzed at least 100 mycytes per animal.

### Quantitative real-time PCR

Total RNA was extracted from 50 mg snap-frozen tissue by homogenization in peqGold Trifast reagent (Peqlab). Residual genomic DNA was removed with Turbo DNAse (Applied Biosystems). The RNA concentration and purity were assayed by spectrophotometry. First-strand cDNA synthesis was performed with random hexamer primer and 1 µg RNA using High Capacity cDNA Reverse Transcription Kit (Applied Biosystems) according to the manufacturer's instruction. (For further detail see Vinon-Zellweger et al [38].)

Sequences from the Ensemble database and the online available Primer3 software [39] were used to create specific, intron-spanning primers for the target genes. Primer pairs were proved for gene-specific amplification and the absence of single nucleotide polymorphisms within binding sites by use of NCBI BLAST tools. Synthesized primers were obtained from Sigma-Aldrich®.

A total of 10 ng cDNA in 5 µl was normally used as template for amplification. An additional 0.5 µl of each primer (5 pmol) and 12.5 µl of Power SYBR Green PCR Master Mix (Applied Biosystems) were added and diluted with water to a volume of 25 µl. The PCR was performed on an Mx3000P thermal cycler (Stratagene). Reaction conditions were 95°C for 10 min, 40 cycles at 95°C for 15 sec, and 60°C for 60 sec, followed by an examination of the melting curve. All samples were analyzed in triplicate. The amplification efficiency of every reaction was checked with the linear regression method [26]. Expression of the gene of interest was divided by the housekeeping gene, glyceraldehyde-3-phosphate dehydrogenase, and expressed as fold-change compared with the corresponding sham-operated rats group.

### Plasma DPP-4 activity and GLP-1 measurements

Blood samples were drawn from the rat retrobulbal venous plexus under isoflurane anesthesia at serial time points immediately before pretreatment and at various time points after the treatment (as described above) up to 72 h postdose. EDTA plasma was frozen for ex vivo measurement of DPP-4 activity. A 20 µl volume of EDTA plasma was diluted with 30 µl of DPP-4 assay buffer (100 mM Tris, 100 mM NaCl, adjusted to pH 7.8 with HCl) and mixed with 50 µl substrate (final concentration 100 µM H-Ala-Pro-7-amido-4-trifluoromethylcoumarin (AlaPro-AFC) was from Bachem) (200 mM stock solution in DMF, diluted 1∶1000 with water). The plate was then incubated at room temperature for 10 min and fluorescence of the wells was determined using a Wallac Victor™ 1420 Multilabel Counter, at an excitation wavelength of 405 nm and an emission wavelength of 535 nm.

Active (uncleaved, 7–36 amide or 7–37) GLP-1 was detected using the commercially available Multi-Array Assay System (K150JWC) from Meso Scale Discovery following the instructions of the supplier. This antibody only detects active GLP-1 (7–36 amide or 7–37 GLP-1) but not cleaved and inactive GLP- 1 (9–36 amide or 9–37 GLP-1).

### Bioanalysis and pharmacokinetic evaluation

Plasma concentrations of linagliptin, sitagliptin and alogliptin were determined by high performance liquid chromatography coupled to tandem mass spectrometry (HPLC-MS/MS) using solid phase extraction for sample preparation with a lower limit of quantification of 0.100 nmol/l for linagliptin and 0.250 nmol/l for sitagliptin and alogliptin. Linagliptin, sitagliptin and alogliptin were analyzed by HPLC-MS/MS assays using [13C3] linagliptin, sitagliptin and alogliptin as internal standards for linagliptin, sitagliptin and alogliptin, respectively. The assays comprise sample clean-up by solid phase extraction (SPE) on SPEC MP3 96-well extraction plates for linagliptin and Focus 96-well extraction plates for sitagliptin and alogliptin. Chromatography for all assays was achieved on a Phenomenex Luna Phenyl-Hexyl 100A, 3 µm, 50×2 mm analytical HPLC column with gradient elution. The analytes were detected and quantified by HPLC-MS/MS using electrospray ionization in the positive ion mode.

Pharmacokinetic analysis was performed using non-compartmental methods with ToxKin V 3.5 (LogicaCMG ITS AG, Switzerland). The linear trapezoidal rule was used for AUC calculation. The terminal half-life (t(1/2)) was calculated as t(1/2) * = *ln2/λ(z) with the terminal elimination rate constant λ(z) as slope of the logarithmic-linear regression line lnC(t)  =  lnC(0 h) – λ(z) × t of two or three data points in the terminal phase. Extrapolated AUC(rest, tz-∞) was calculated according to Aronson et al [40], using the calculated concentration and time (t(z)) of the last measurable data point.

### Statistics

For PCR analysis, means ± SEM were compared using the two-tailed Student's t-test for two groups and one-way analysis of variance with paired comparisons for more than two groups. Correlation analysis was performed using Spearman's rank correlation test. P-values of less than 0.05 were considered significant. These statistical tests were performed using SPSS 10.0 for Windows (SPSS Inc., Chicago, IL, USA). For AUC determinations, statistical comparison between sham and 5/6 nephrectomy was done by a one-factorial analysis of variance (ANOVA) with heteroscedastic variances. For kidney function analysis before and after drug administration for each parameter the values were analyzed separately using an analysis of variance for repeated measurements, with factors experimental group and time as fixed effects. The values belonging to the same animal are modeled as repeated measurements assuming an unstructured covariance matrix.

### Analysis of kidney function parameters before and after compound administration

For each parameter, the log10-transformed values were analyzed separately using an ANOVA for repeated measurements with factors for experimental group and time as fixed effects. The log10-transformed values belonging to the same animal are modeled as repeated measurements assuming an unstructured covariance matrix. These statistical analyses were carried out with the software product SAS (SAS Institute, Cary, NC USA), version 9.2.

## References

[1] Foley RN, Murray AM, Li S, Herzog CA, McBean AM (2005). Chronic kidney disease and the risk for cardiovascular disease, renal replacement, and death in the United States Medicare population, 1998 to 1999.. J Am Soc Nephrol.

[2] Go AS, Chertow GM, Fan D, McCulloch CE, Hsu CY (2004). Chronic kidney disease and the risks of death, cardiovascular events, and hospitalization.. N Engl J Med.

[3] Tonelli M, Wiebe N, Culleton B, House A, Rabbat C (2006). Chronic kidney disease and mortality risk: a systematic review.. J Am Soc Nephrol.

[4] Ossum A, van Deurs U, Engstrom T, Jensen JS, Treiman M (2009). The cardioprotective and inotropic components of the postconditioning effects of GLP-1 and GLP-1(9-36)a in an isolated rat heart.. Pharmacol Res.

[5] Best JH, Hoogwerf BJ, Herman WH, Pelletier EM, Smith DB (2011). Risk of cardiovascular disease events in patients with type 2 diabetes prescribed the glucagon-like peptide 1 (GLP-1) receptor agonist exenatide twice daily or other glucose-lowering therapies: a retrospective analysis of the LifeLink database.. Diabetes Care.

[6] Grieve DJ, Cassidy RS, Green BD (2009). Emerging cardiovascular actions of the incretin hormone glucagon-like peptide-1: potential therapeutic benefits beyond glycaemic control?. Br J Pharmacol.

[7] Herman GA, Stevens C, Van Dyck K, Bergman A, Yi B (2005). Pharmacokinetics and pharmacodynamics of sitagliptin, an inhibitor of dipeptidyl peptidase IV, in healthy subjects: results from two randomized, double-blind, placebo-controlled studies with single oral doses.. Clin Pharmacol Ther.

[8] Bergman AJ, Stevens C, Zhou Y, Yi B, Laethem M (2006). Pharmacokinetic and pharmacodynamic properties of multiple oral doses of sitagliptin, a dipeptidyl peptidase-IV inhibitor: a double-blind, randomized, placebo-controlled study in healthy male volunteers.. Clin Ther.

[9] Weir GC, Mojsov S, Hendrick GK, Habener JF (1989). Glucagonlike peptide I (7-37) actions on endocrine pancreas.. Diabetes.

[10] Scheen AJ (2010). Pharmacokinetics of dipeptidylpeptidase-4 inhibitors.. Diabetes Obes Metab.

[11] Shvarts V (2008). [New avenues for pharmacotherapy of type 2 diabetes mellitus].. Klin Med (Mosk).

[12] Blech S, Ludwig-Schwellinger E, Grafe-Mody EU, Withopf B, Wagner K (2010). The metabolism and disposition of the oral dipeptidyl peptidase-4 inhibitor, linagliptin, in humans.. Drug Metab Dispos.

[13] Heise T, Graefe-Mody EU, Huttner S, Ring A, Trommeshauser D (2009). Pharmacokinetics, pharmacodynamics and tolerability of multiple oral doses of linagliptin, a dipeptidyl peptidase-4 inhibitor in male type 2 diabetes patients.. Diabetes Obes Metab.

[14] Yan X, Sano M, Lu L, Wang W, Zhang Q (2010). Plasma concentrations of osteopontin, but not thrombin-cleaved osteopontin, are associated with the presence and severity of nephropathy and coronary artery disease in patients with type 2 diabetes mellitus.. Cardiovasc Diabetol.

[15] Kujal P, Vernerova Z (2008). [5/6 nephrectomy as an experimental model of chronic renal failure and adaptation to reduced nephron number].. Cesk Fysiol.

[16] Horiba N, Masuda S, Takeuchi A, Saito H, Okuda M (2004). Gene expression variance based on random sequencing in rat remnant kidney.. Kidney Int.

[17] Masuda S (2003). Functional characteristics and pharmacokinetic significance of kidney-specific organic anion transporters, OAT-K1 and OAT-K2, in the urinary excretion of anionic drugs.. Drug Metab Pharmacokinet.

[18] Kirino Y, Sato Y, Kamimoto T, Kawazoe K, Minakuchi K (2009). Interrelationship of dipeptidyl peptidase IV (DPP4) with the development of diabetes, dyslipidaemia and nephropathy: a streptozotocin-induced model using wild-type and DPP4-deficient rats.. J Endocrinol.

[19] Pucci L, Triscornia S, Lucchesi D, Fotino C, Pellegrini G (2007). Cystatin C and estimates of renal function: searching for a better measure of kidney function in diabetic patients.. Clin Chem.

[20] Rigalleau V, Beauvieux MC, Le Moigne F, Lasseur C, Chauveau P (2008). Cystatin C improves the diagnosis and stratification of chronic kidney disease, and the estimation of glomerular filtration rate in diabetes.. Diabetes Metab.

[21] Willems D, Wolff F, Mekhali F, Gillet C (2009). Cystatin C for early detection of renal impairment in diabetes.. Clin Biochem.

[22] Amann K, Breitbach M, Ritz E, Mall G (1998). Myocyte/capillary mismatch in the heart of uremic patients.. J Am Soc Nephrol.

[23] Mall G, Huther W, Schneider J, Lundin P, Ritz E (1990). Diffuse intermyocardiocytic fibrosis in uraemic patients.. Nephrol Dial Transplant.

[24] Tyralla K, Amann K (2003). Morphology of the heart and arteries in renal failure.. Kidney Int Suppl.

[25] Lindsay MM, Maxwell P, Dunn FG (2002). TIMP-1: a marker of left ventricular diastolic dysfunction and fibrosis in hypertension.. Hypertension.

[26] Rabkin R, Awwad I, Chen Y, Ashley EA, Sun D (2008). Low-dose growth hormone is cardioprotective in uremia.. J Am Soc Nephrol.

[27] Tian J, Shidyak A, Periyasamy SM, Haller S, Taleb M (2009). Spironolactone attenuates experimental uremic cardiomyopathy by antagonizing marinobufagenin.. Hypertension.

[28] Boerrigter G, Costello-Boerrigter LC, Harty GJ, Lapp H, Burnett JC (2007). Des-serine-proline brain natriuretic peptide 3-32 in cardiorenal regulation.. Am J Physiol Regul Integr Comp Physiol.

[29] Mizobuchi M, Towler D, Slatopolsky E (2009). Vascular calcification: the killer of patients with chronic kidney disease.. J Am Soc Nephrol.

[30] Senkel S, Lucas B, Klein-Hitpass L, Ryffel GU (2005). Identification of target genes of the transcription factor HNF1beta and HNF1alpha in a human embryonic kidney cell line.. Biochim Biophys Acta.

[31] Ku HC, Chen WP, Su MJ (2010). GLP-1 signaling preserves cardiac function in endotoxemic Fischer 344 and DPP4-deficient rats.. Naunyn Schmiedebergs Arch Pharmacol.

[32] Meier JJ, Nauck MA, Kranz D, Holst JJ, Deacon CF (2004). Secretion, degradation, and elimination of glucagon-like peptide 1 and gastric inhibitory polypeptide in patients with chronic renal insufficiency and healthy control subjects.. Diabetes.

[33] Amori RE, Lau J, Pittas AG (2007). Efficacy and safety of incretin therapy in type 2 diabetes: systematic review and meta-analysis.. Jama.

[34] Pfisterer M, Buser P, Rickli H, Gutmann M, Erne P (2009). BNP-guided vs symptom-guided heart failure therapy: the Trial of Intensified vs Standard Medical Therapy in Elderly Patients With Congestive Heart Failure (TIME-CHF) randomized trial.. Jama.

[35] National Institute of Diabetes and Digestive and Kidney Diseases US Renal Data Systems: USRDS 2011 annual data report: Atlas of End Stage Renal Disease in the United States. National Institutes of Health, Bethesda, Maryland, USA; http://www.usrds.org/adr.aspx

[36] Kalk P, Eggert B, Relle K, Godes M, Heiden S (2007). The adenosine A1 receptor antagonist SLV320 reduces myocardial fibrosis in rats with 5/6 nephrectomy without affecting blood pressure.. Br J Pharmacol.

[37] Kalk P, Godes M, Relle K, Rothkegel C, Hucke A (2006). NO-independent activation of soluble guanylate cyclase prevents disease progression in rats with 5/6 nephrectomy.. Br J Pharmacol.

[38] Vignon-Zellweger N, Relle K, Kienlen E, Alter M, Seider P (2011). Endothelin-1 overexpression restores diastolic function in eNOS knockout mice.. J Hypertens.

[39] Rozen S, Skaletsky H (2000). Primer3 on the WWW for general users and for biologist programmers.. Methods Mol Biol.

[40] Aronson JK, Dengler HJ, Dettli L, Follath F (1988). Standardization of symbols in clinical pharmacology.. Eur J Clin Pharmacol.

